# Supplementary material for: Broadband Multidimensional Spectroscopy Identifies the Amide II Vibrations in Silkworm Films
Source: Molecules. 2022 Sep 23;27(19):6275. doi: 10.3390/molecules27196275 (PMC9571984; doi:10.3390/molecules27196275)
Supplement: Supplementary file 1 [file molecules-27-06275-s001.zip › molecules-1888581-supplementary.pdf]

Supplementary Information

Broadband multidimensional spectroscopy identifies the amide II vibrations in silkworm films

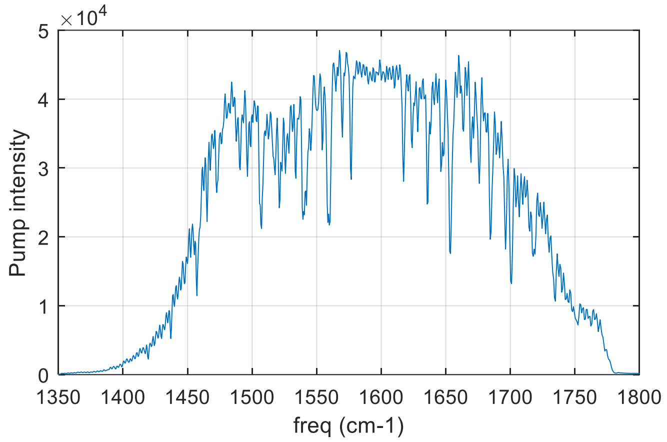

Fig S1: the spectrum of the laser pulse used to record these spectra. The dips in this spectrum are due to water absorption lines inside our unpurged monochromator – they are not present at the point of interaction with the sample.

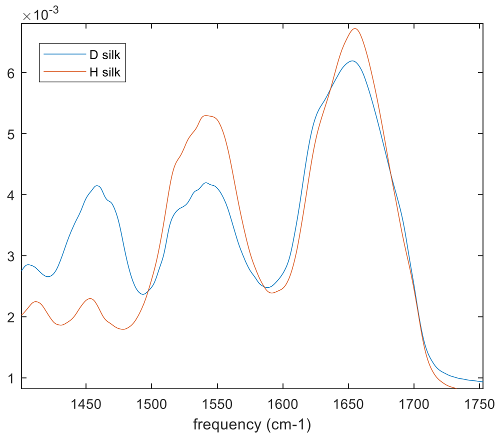

Fig S2: the FTIR spectra of silk before and after deuteration. Deuteration shifts weight from the amide II to the amide II′ region, however there is already some intensity in that region due to sidechain modes.

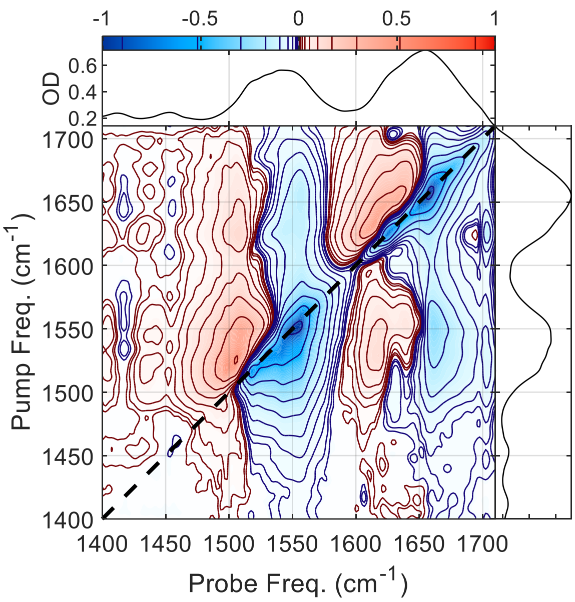

Fig S3: 2D spectrum before deuteration, with the FTIR spectrum plotted alongside. The amide I/II cross-peaks are much less resolved than in the deuterated spectrum, although the general structure of the cross-peak regions is the same.

Amide II′ cross peak regions

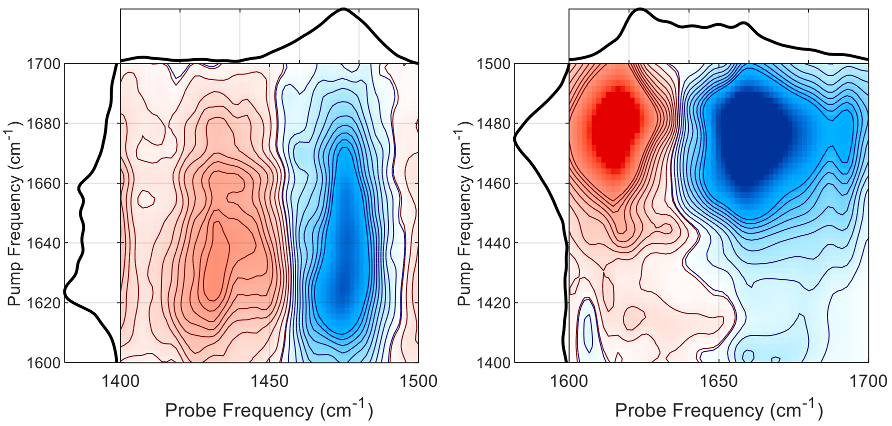

Fig S4: (a & b) Zoom of the amide I′/II′ downward (a) and upwards (b) cross peak regions. Compared to Fig. 2, the color and contour scale is enhanced 10x, so that weak features are more easily discerned. The diagonal cut is shown alongside both 2DIR spectra.

We can also attempt a similar cross-peak analysis to assign the (N-D) amide II′ region. Zooms of the two cross-peak sectors are shown in Fig. S4. Unfortunately, due to our limited laser bandwidth we can only resolve the peak at around 1475 cm^{-1} in our 2DIR spectra. Additionally, the presence of many sidechain modes in this region complicates spectral analysis. Despite this, we can make some qualitative statements about the amide II′ spectrum. In the (I/II′) downwards cross peak region (Fig. S4a), we can see that the 1480 cm^{-1} cross-peak that extends throughout the amide I region, although the intensity is somewhat higher at 1620 cm^{-1}.

We can thus conclude that both β-sheets and α-helix modes contribute here. This assignment is reinforced by the upwards (II'/I) cross peak region (Fig. S4b), where we see a clear cross-peak at (1475, 1695) cm^{-1} along with the (1475, 1660) cm^{-1} peak. Since the 1695 cm^{-1} peak is very weak, for it to be visible at all implies that the 1475 cm^{-1} peak carries significant β-sheet character. Note that excited state absorption obscures any cross peak with the 1620 cm^{-1} peak. We cannot make any stronger statements on the assignment of the amide II' region; we intend to revisit this problem with a laser bandwidth tuned to investigating this region.
